# Soluble BTN2A1 Is a Potential Prognosis Biomarker in Pre-Treated Advanced Renal Cell Carcinoma

**DOI:** 10.3389/fimmu.2021.670827

**Published:** 2021-04-20

**Authors:** Emilien Billon, Brice Chanez, Philippe Rochigneux, Laurence Albiges, Cécile Vicier, Géraldine Pignot, Jochen Walz, Anne-Sophie Chretien, Gwenaelle Gravis, Daniel Olive

**Affiliations:** ^1^ Team Immunity and Cancer, Centre de Recherche en Cancérologie de Marseille (CRCM), Inserm, U1068, CNRS, UMR7258, Institut Paoli-Calmettes, Aix-Marseille University, UM105, Marseille, France; ^2^ Immunomonitoring Department, Institut Paoli-Calmettes, Marseille, France; ^3^ Department of Medical Oncology, Institut Paoli-Calmettes, Marseille, France; ^4^ Department of Cancer Medicine, Institut Gustave Roussy, Villejuif, France; ^5^ Department of Urology, Institut Paoli-Calmettes, Marseille, France

**Keywords:** renal cell carcinoma, butyrophilin, nivolumab, immunotherapy, γδ T cells

## Abstract

The development of immune checkpoint inhibitors (ICI) has dramatically changed the landscape of therapies for metastatic renal cell carcinoma. However, many patients do not benefit from such therapy and prognostic or predictive validated biomarker validated for ICI are still needed to better select and treat patient. Plasmatic soluble immune checkpoints have been described as potential immune biomarkers in hematological malignancies and solids tumors, then, we would like to explore the prognostic value of different soluble immune checkpoints in patients with mRCC treated with nivolumab after TKI. We prospectively collected plasma samples before nivolumab infusion from 38 patients previously treated for mRCC with TKI at Paoli-Calmettes Institute, from the NIVOREN GETUG-AFU 26 study (NCT03013335). Enzyme-linked immunosorbent assays (ELISA) were performed for soluble forms of PD-1, PD-L1, global BTN3, BTLA, BTN3A1 and BTN2A1. Among the different soluble checkpoints analyzed, only high baseline plasmatic level of BTN2A1 was significantly associated with shorter PFS: median PFS was 3.95 months for sBTN2A1high vs 14.30 months for sBTN2A1low (sBTN2A1 cut-off: 6.7ng/mL; HR = 2.26, 95%CI [0.68 – 4.60], p = 0.0307). There was no statistical difference in OS between sBTN2A1high and sBTN2A1^low^. Our results suggest that the baseline level of plasmatic BTN2A1 could be an independent prognosis factor of PFS after nivolumab for pre-treated patient with mRCC. However, these results need to be validated in a larger prospective cohort and the biological role of BTN subfamily and γδ T cell immunity in mRCC must be elucidated.

## Introduction

The development of immune checkpoint inhibitors (ICI) has dramatically changed the landscape of therapies for metastatic renal cell carcinoma (mRCC), as such in first line associated to tyrosine kinase inhibitors (TKI) (1) or as monotherapy (2). However, many patients do not benefit from such therapy and prognostic or predictive validated biomarker validated for ICI are still needed to better select and treat patient according to the best genomic profiles. The International Metastatic RCC Database Consortium (IMDC) prognostic model (3) is used to predict survival and adapted treatment strategy of untreated mRCC patients and was validated for patients previously treated with TKI, before second line TKI. Identifying new prognostic biomarkers in this population is needed to improve treatment strategy for mRCC patients.

Plasmatic soluble immune checkpoints, such as programmed death protein (PD-1) with its ligands PD-L1 and PD-L2, the B7/butyrophilin-like receptors such as butyrophilin sub-family 3A (CD277) members (BTN3A) and butyrophilin sub-family 2A (CD209) members, and the B and T lymphocyte attenuator (BTLA) have been described as potential immune biomarkers in hematological malignancies (4) and solids tumors (pancreas (5), gastric (6), lung cancers (7) and hepatocarcinoma (8)). In localized RCC, sBTLA was associated with poorer relapse-free survival and overall survival (OS) after nephrectomy (9).

We aimed to explore the prognostic value of different soluble immune checkpoints in mRCC treated with nivolumab after TKI.

## sBTN2A1 Is a Prognostic in Patients With Pre-Treated Advanced Renal Cell Carcinoma

To explore the prognostic value of different soluble immune checkpoints, we prospectively collected plasma samples before nivolumab infusion from patients previously treated for mRCC with TKI at Paoli-Calmettes Institute (Nivo-Rein cohort), from the NIVOREN GETUG-AFU 26 study (NCT03013335). Enzyme-linked immunosorbent assays (ELISA) were performed for soluble forms of PD-1, PD-L1, global BTN3, BTLA, BTN3A1 and BTN2A1. All patients provided informed consent prior to enrolment. The receiver operating characteristic (ROC) curves were generated for each marker. The areas under the curves (AUC) were assessed to evaluate each marker performance for discriminating short term responder (patients with PFS > median) from long-term responder (patients with PFS < median). Cut-off was selected to obtain the best sensitivity and specificity for discriminating long-term responders from short term responders. Survival analyses were performed using the Kaplan–Meier method and log-rank test. Multivariate analysis of survival was performed using the Cox-regression model, including all variables associated with progression-free survival (PFS) (p < 0.1).

**Figure 1:**
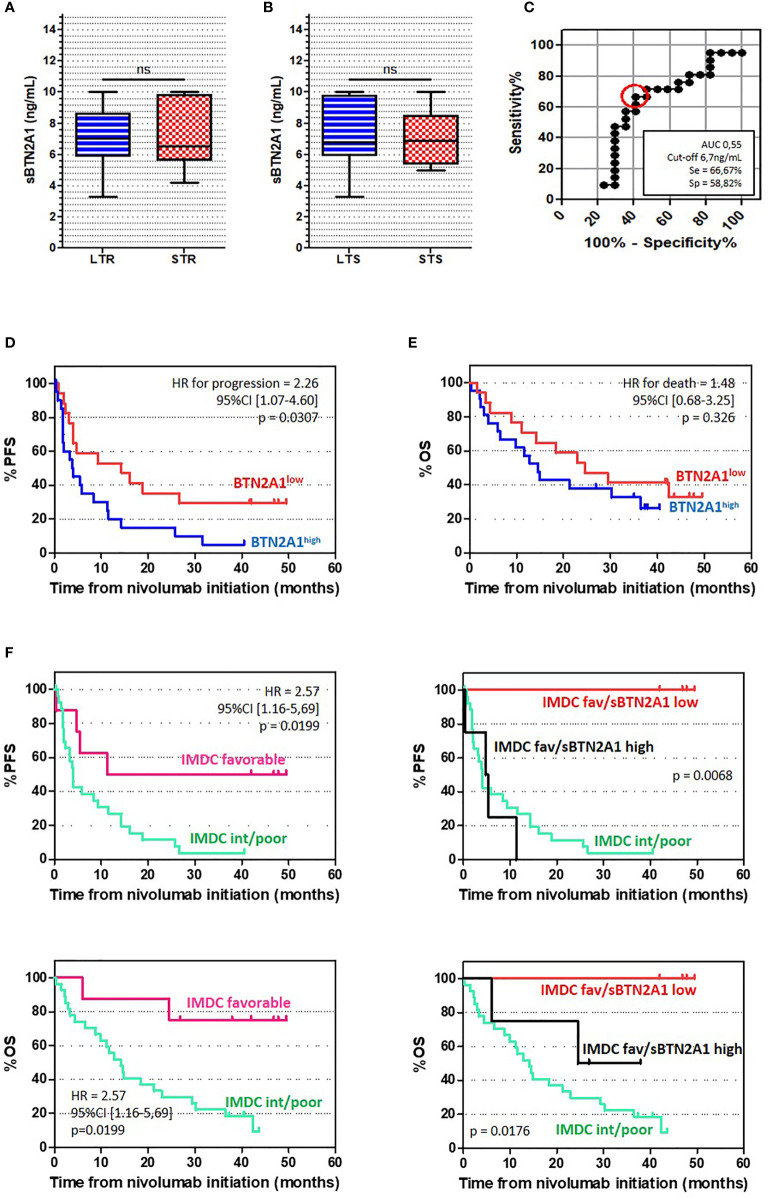
sBTN2A1 seemed to be an independent prognostic factor for PFS in patients with mRCC **(A, B)** sBTN2A1 expression according to the time of response **(A)** and the time of overall survival **(B)**, assessed par enzyme linked immunosorbent assay. Paired-t test was used to compare differences between the groups. LTR, Long term responders; patients with PFS > median; STR, short term responders; patients with PFS < median; LTS, Long term survival; patients with OS > median; STS, Short term survival; patients with OS < median. **(C)** Receiver operating characteristics (ROC) curve analysis of plasma level for sBTN2A1. ROC curve was plotted for sensitivity and specificity of time of response. **(D, E)** Kaplan Meier analysis of PFS **(D)** and OS **(E)** in patients with high and low plasma levels of BTN2A1. **(F)** Kaplan Meier analysis of PFS (top) and OS (bottom) according to the IMDC prognosis subgroup and sBTN2A1 level.

We included 38 patients between March 2016 and November 2017. Patients’ characteristics are summarized in **Table 1**. With a median follow-up of 19.8 months, 31 (82%) patients had tumor progression and 25 (66%) died. Among the different soluble checkpoints analyzed, only high baseline plasmatic level of BTN2A1 was significantly associated with shorter PFS. Cut-off retained was 6.7ng/mL, that approximate the median value of 6.8ng/mL. There was no significant difference between patients with sBTN2A1^low^ and sBTN2A1^high^ level; excepted for age (**Table 1**). Median PFS was 3.95 months for sBTN2A1^high^ *vs.* 14.30 months for sBTN2A1^low^ (**Figures 1A–C**) (HR = 2.26, 95%CI [0.68 – 4.60], p = 0.0307). There was no statistical difference in OS between sBTN2A1^high^ and sBTN2A1^low^ (median OS = 14.6 *vs.* 24.5 months, respectively, p = 0.326) (**Figures 1D, E**). There was no significant impact on survival of plasmatic level of BTLA (median PFS for BTLA^high^ 14.3 vs. 7.1 months, HR = 0.57, 95%CI [0.22 – 1.51]; cut-off: 4.33ng/mL), PD-L1 (median PFS for PD-L1^high^ 12.9 vs. 4.1 months, HR = 0.79, 95%CI [0.38 – 1.65]; cut-off: 0.88ng/mL), PD-1 (median PFS for PD-1^high^ 12.9 vs. 4.0 months, HR = 0.59, 95%CI [0.26 – 1.35; cut-off: 0.83ng/mL]), or BTN3A1 (median PFS for BTN3A1^high^ 9.3 vs. 4.7 months, HR = 0.68, 95%CI [0.22 – 2.05]; cut-off: 1.55ng/mL) in this cohort (**Supplementary Figure 1**).

**Table 1:** Baseline characteristics (n = 38).

	Entire cohort	sBTN2A1 low (n = 17)	sBTN2A1 high (n = 21)	p
Median age (range) – years	60,5 (38 - 81)	54,4 (38,8 - 71,5)	65,3 (51,2 - 81,4)	*0,002*
Sex, n (%)				*0,132*
Male	32 (84,2)	16 (94,1)	16 (76,2)	
Female	6 (15,8)	1 (5,9)	5 (23,8)	
Histological classification				*0,679*
Clear cell	35 (92,1)	16 (94,1)	19 (90,5)	
Other	3 (7,9)	1 (5,9)	2 (9,5)	
Number of metastasis site, n (%)				*0,658*
≤2	12 (31,6)	6 (35,3)	6 (28,6)	
>2	26 (38,4)	11 (64,7)	15 (71,4)	
IMDC risk subroup				*0,699*
Favorable	8 (22,9)	5 (31,3)	3 (15,8)	
Intermediate	18 (51,4)	7 (43,7)	11 (57,9)	
Poor	9 (25,7)	4 (25,0)	5 (26,3)	
Undefined	3	1	2	
Karnofsky performans status, n (%)				*0,635*
≥80%	23 (60,5)	11 (64,7)	12 (57,1)	
<80%	15 (39,5)	6 (35,3)	9 (42,9)	
Hemoglobin rate, n (%)				*0,13*
≥lower limit of normal	9 (23,7)	6 (35,3)	3 (14,3)	
<lower limit of normal	29 (76,3)	11 (64,7)	18 (85,7)	
Calcium > upper limit of normal				*0,16*
≥upper limit of normal	28 (80)	15 (93,8)	13 (68,4)	
>upper limit of normal	7 (20)	1 (6,2)	6 (31,6)	
Unknown	3	1	2	
Platelets > upper limit of normal				*0,038*
≥upper limit of normal	32 (84,2)	12 (70,6)	20 (95,2)	
>upper limit of normal	6 (15,8)	5 (29,4)	1 (4,8)	
Neutrophil > upper limit of normal				*0,189*
≥upper limit of normal	35 (94,6)	15 (88,2)	20 (100)	
>upper limit of normal	2 (5,4)	2 (11,8)	0 (0,0)	
Unknown	1	0	1	
Median time from the diagnosis of metastatic disease, months (range)	17,6 (1,5 - 87,2)	17,3 (3,0 - 64,6)	31,2 (1,5 - 87,2)	*0,061*

In univariate analysis, PFS was associated with baseline anemia, IMDC subgroups and sBTN2A1 level (**Table 2**). sBTN2A1 was associated with PFS in multivariate analysis (HR = 2.80, 95%CI [1.22-6.41], p=0.015) (**Table 3**). sBTN2A1 was able to discriminate two different prognostic groups among the subgroup of patients with favorable prognosis according to the IMDC model (**Figure 1F**): favorable IMDC/sBTN2A1^high^ patients had lower PFS than favorable IMDC/sBTN2A1^low^ patients, close to poor/intermediate IMDC patients (median PFS = 5.1months vs. ND, respectively, HR =18.1, p= 0.0067).

**Table 2:** Univariate hazard ratio for PFS and OS.

Variables	Progression free survival			Overall survival		
	Hazard ratio	95%IC	*P*	Hazard ratio	95%IC	*P*
Age > median	0,93	0,46 - 1,89	*0,840*	0,83	0,38 - 1,81	*0,634*
Sex (male)	1,33	0,51 - 3,48	*0,564*	1,18	0,41 - 3,43	*0,762*
KPS < 80%	1,87	0,87 - 4,02	*0,098*	2,64	1,13 - 6,18	*0,025*
Hemoglobin < lower limit of normal	2,27	1,06 - 4,86	*0,034*	2,62	1,14 - 6,04	*0,024*
Calcium > upper limit of normal	0,99	0,38 - 2,63	*0,990*	1,85	0,63 - 5,43	*0,265*
Platelets > upper limit of normal	1,03	0,39 - 2,74	*0,947*	1,39	0,47 - 4,09	*0,546*
Neutrophil > upper limit of normal	1,41	0,27 - 7,39	*0,683*	11,63	0,86 - 155,8	*0,064*
IMDC risk subgroup						
Favorable	1,00			1,00		
Intermediate	3,54	1,16 - 10,79	*0,026*	5,74	1,30 - 25,33	*0,021*
Poor	2,98	0,89 - 10,01	*0,077*	6,92	1,44 - 33,27	*0,016*
sBTN2A1 high	2,26	0,68 – 4,60	*0,031*	1,48	0,68 - 3,25	*0,326*

KPS, Karnofsky performans status; IMDC, International Metastatic RCC Database Consortium.

**Table 3:** Multivariate hazard ratio for PFS and OS.

Variables	Progression free survival			Overall survival		
	Hazard ratio	95%IC	*P*	Hazard ratio	95%IC	*P*
Age > median	0,42	0,00 - 0,99	*0,047*	0,46	0,20 - 1,05	*0,065*
Sex (male)	2,09	0,71 - 6,13	*0,180*	2,02	0,65 - 6,27	*0,224*
IMDC risk subgroup						
Favorable	1,00			1,00		
Intermediate	4,87	1,46 - 16,23	*0,010*	8,03	1,73 - 37,27	*0,008*
Poor	4,46	1,12 - 17,75	*0,034*	12,06	2,26 - 64,18	*0,004*
sBTN2A1 high	2,80	1,22 - 6,41	*0,015*	1,33	0,58 - 3,08	*0,500*

IMDC, International Metastatic RCC Database Consortium.

## Discussion

To our knowledge, this study is the first that described sBTN2A1 as potential prognostic biomarker in patients with pre-treated mRCC. In the previous NIVOREN GETUG-AFU 26 study, it was reported that tissue PD-1 and AXL expression by immunohistochemistry were associated with PFS and confirmed that PBRM-1 loss was a strong prognostic factor (10). Incorvaia et al. explored the prognostic value of soluble forms of PD-1, PD-L1, global BTN3, BTN3A1 and BTN2A1 in a prospective cohort of metastatic renal cell carcinoma patients (11). They identified sPD-1, sPD-L1 and sBTN3A1 as potential biomarkers. However, our study did not significantly confirm these results, with only a tendency for sPD-1 and sPD-L1 and this difference could be explained by the small number of patients in our cohort. Conversely, sBTN2A1 was not significantly associated with survival in their study but patients with PFS > 18 months seemed to have lower level of sBTN2A1 than patients with PFS < 18 months. The small number of patients in their learning cohort (n=21) could also explain the difference between the two works; and reinforce the necessity to validate these results in a larger prospective cohort.

The BTN sub-family plays an important role in V_ϒ_9V_δ_2 T-cells activation and regulation by direct or indirect presentation of self and nonself phosphoantigens. The cell surface molecule BTN2A1 binds to the γδ TCR, in conjunction with BTN3A1, and signals the presence of phosphoantigens to γδ T cells (12). Gamma delta T (γδT) lymphocytes are members of the immune system which display both innate and adaptive functions (13). They recognize low molecular mass nonpeptide ligands named “phosphoantigens” that comprise isoprenoid pathway metabolites, as (E)-4-hydroxy-3-methyl-but-2-enyl PPi (HMB-PP) derived from bacteria and protozoa (14) and isopentenyl PPi (IPP) derived from host cells (15). There are evidence of their cytotoxic activity against tumor cells in certain cancers (16), and they are able to recognize tumor cells without the need for major histocompatibility complex (MHC) antigen presentation. However, there also evidence that suggested that γδT lymphocytes may also have a pro-tumorigenic role (17), by impairing the function of other antitumor immune cells or enhancing immunosuppressive cell function such as MDSCs.

We can hypothesize that high level of circulating BTN2A1 could lead to Vγ9Vδ2 T-cells exhaustion that facilitate tumor immune escape mechanisms (18), and/or activate Vγ9Vδ2 T cells with pro-tumorigenic characteristics, and explain the poorer outcomes of patients with high level of circulating BTN2A1. These Vγ9Vδ2 T-cells alterations could also impaired anti-PD-1 activities on these cells and then, impaired clinical efficacy of anti-PD-1 agents in patients with mRCC.

Soluble BTN2A1 could be a new approach to predict resistance and avoiding toxicities for mRCC treated by nivolumab. Targeting BTN sub-family molecule could represent a new therapy approach for mRCC patients. Despite the small number of patients in this cohort, our results suggest that the baseline level of plasmatic BTN2A1 could be an independent prognosis factor of PFS after nivolumab for pre-treated patient with mRCC. However, these results need to be validated in a larger prospective cohort and the biological role of BTN subfamily and γδ T cell immunity in mRCC must be elucidated.

## Data Availability Statement

The raw data supporting the conclusions of this article will be made available by the authors, without undue reservation.

## Ethics Statement

The studies involving human participants were reviewed and approved by Institut Paoli Calmettes. The patients/participants provided their written informed consent to participate in this study.

## Author Contributions

Conception and design: DO, GG. Acquisition of data: EB and BC. Analysis and interpretation of data: EB, BC, and DO. Drafting of the manuscript: EB, PR, and DO. Critical revision of the manuscript for important intellectual content: EB, BC, PR, LA, CV, GP, JW, A-SC, GG, and DO. Statistical analysis: EB. Administrative, technical, or material support: DO. Supervision: DO and GG. All authors contributed to the article and approved the submitted version.

## Funding

DO is supported by research grant of FRM (Equipe FRM 2018 DEQ20180339209) and RHU Pioneer. EB is supported by ITMO Cancer of Aviesan on funds administered by Inserm.

## Conflict of Interest

The authors declare that the research was conducted in the absence of any commercial or financial relationships that could be construed as a potential conflict of interest.

## Abbreviations

BTLA, B and T lymphocyte attenuator; BTN, butyrophilin; CI, confidence interval; ELISA, Enzyme-linked immunosorbent assays; HR, hazard ratio; ICI, immune checkpoint inhibitors; IMDC, International Metastatic RCC Database Consortium; mRCC, metastatic renal cell carcinoma; OS, overall survival; PD-1, programmed death protein 1; PDL-1, programmed death ligand 1; PFS, progression-free survival; TKI, tyrosine kinase inhibitor.
