# Supplementary figures and images for: Soluble BTN2A1 Is a Potential Prognosis Biomarker in Pre-Treated Advanced Renal Cell Carcinoma
Source: Front Immunol. 2021 Apr 20;12:670827. doi: 10.3389/fimmu.2021.670827 (PMC8096349; doi:10.3389/fimmu.2021.670827)

## Slide 1
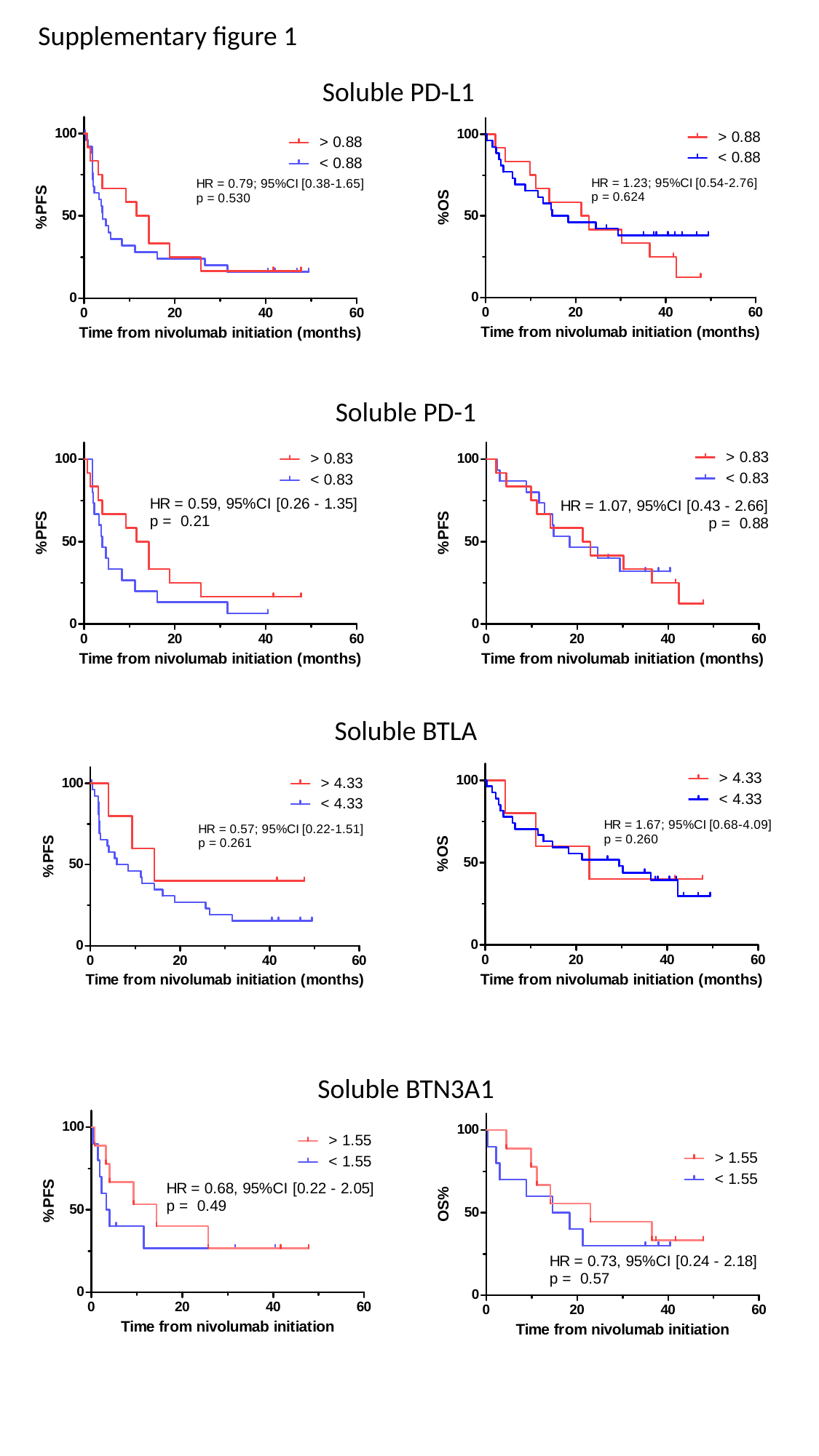

Supplementary figure 1
Soluble PD-L1
Soluble PD-1
Soluble BTLA
Soluble BTN3A1

Supplement: Supplementary file 1 [file Presentation_1.pptx]
